# A Conjugated Aptamer-Gold Nanoparticle Fluorescent Probe for Highly Sensitive Detection of rHuEPO-α

**DOI:** 10.3390/s111110490

**Published:** 2011-11-03

**Authors:** Jiefang Sun, Aitao Guo, Zhaoyang Zhang, Lei Guo, Jianwei Xie

**Affiliations:** 1 Beijing Institute of Pharmacology and Toxicology, No. 27, Taiping Road, Beijing 100850, China; E-Mails: sunjf2001@163.com (J.S.); zyzhang2009@gmail.com (Z.Z.); 2 Department of Pathology, General Hospital of the Chinese People’s Liberation Army, No. 28, Fuxing Road, Beijing 100853, China; E-Mail: aitguo@hotmail.com (A.G.)

**Keywords:** aptamers, gold nanoparticles, rHuEPO-α, fluorescent probe

## Abstract

We present here a novel conjugated aptamer-gold nanoparticle (Apt-AuNPs) fluorescent probe and its application for specific detection of recombinant human erythropoietin-α (rHuEPO-α). In this nanobiosensor, 12 nm AuNPs function as both a nano-scaffold and a nano-quencher (fluorescent energy acceptor), on the surface of which the complementary sequences are linked (as cODN-AuNPs) and pre-hybridized with carboxymethylfluorescein (FAM)-labeled anti-rHuEPO-α aptamers. Upon target protein binding, the aptamers can be released from the AuNP surface and the fluorescence signal is restored. Key variables such as the length of linker, the hybridization site and length have been designed and optimized. Full performance evaluation including sensitivity, linear range and interference substances are also described. This nanobiosensor provides a promising approach for a simple and direct quantification of rHuEPO-α concentrations as low as 0.92 nM within a few hours.

## 1. Introduction

Gold nanoparticles (AuNPs), as a class of nanomaterials with many unique properties such as colorimetric, conductivity, and nonlinear optical properties, as well easy functionalization with biological recognition elements, hold great promise for biological and medicinal applications [1–3]. AuNPs functionalized with oligonucleotides (ODN-AuNPs) have emerged as a kind of novel nanomaterial for diagnosis, therapy [4] and materials design [5]. Taking advantage of the highly efficient fluorescence quenching properties of AuNPs for proximately fluorescent dyes through energy-transfer processes [6], the hybridization/recognition ability of ODN-AuNPs has been successfully employed to construct various sensitive and effective sensing probes [7,8]. Importantly, detection methods relying on ODN-AuNPs show more sensitivity than that of many ODN associated molecular probes commonly used in conventional assays. Some ODN-AuNPs based strategies have been approved by US FDA and commercialized recently [9].

Aptamers are oligonucleotides which are generated by *in vitro* systematic evolution of ligands by exponential enrichment (SELEX) procedures [10,11]. They exhibit high affinity and selectivity for various target molecules (metal ions, peptides, proteins and even intact cells), and are thus considered to be a kind of attractive, excellent recognition module. With the progress of nanobiotechnology, aptamers have been promoted as ideal diagnostic reagents and potential antibody alternatives for the development of biomolecular nanosensors [12–14]. Huang *et al.* [15] have developed an aptamer-functionalized AuNPs probe for a sensitive analysis of platelet derived growth factor by monitoring the fluorescent resonance energy transfer (FRET) process between AuNPs and an intercalating dye DMDAP. The fluorescence of DMDAP was quenched efficiently by AuNPs when it intercalated with the aptamer on the surface of AuNPs, but restored when aptamer bound with its target protein and released DMDAP. Mirkin *et al.* [16] have also demonstrated a kind of aptamer bound AuNPs probe. After being hybridized with a short, fluorophore-labeled complementary oligonucleotide as the signal sequence, this aptamer-AuNPs conjugate could act as an effective intracellular nano-flare probe. If ATP target was present, it would interact with aptamers on the surface of AuNPs with high specificity and then the enhanced fluorescence signal that correlated with the presence and abundance of intracellular ATP levels in live samples was shown. In these strategies, aptamers are directly linked onto the surface of AuNPs through metal affinity of thiol group to gold. Unfortunately, the recognition ability of aptamers is affected to some extent compared with its status in free solution, as evidenced by a slower diffusion rate of DMDAP to the aptamer-AuNPs surface [15] or decreased binding constants [16]. The structural conformation of aptamers is not so readily formed when they are restricted by being tethered on the solid surface of AuNPs.

Recently, Fan *et al.* [17] have developed aptamer-based multicolor fluorescent AuNPs probes, in which the complementary counterparts are assembled at the surface of the AuNPs, and then hybridized with aptamer to form duplexes for multiplex detection of small molecules, *i.e.*, adenosine, potassium, and cocaine, respectively. This proof of concept indicates some feasibility of macrobiomolecule detection since such a design makes aptamers more flexible than when directly linked to AuNPs, which could help to maintain the full recognition ability of aptamers for their target proteins at low concentration. In this work, we propose a further research based on Fan *et al.*’s [17] results and apply such a strategy to protein, in which the complementary aptamer oligonucleotides are directly linked onto the surface of AuNPs (cODN-AuNPs), the aptamers are then hybridized with these cODN-AuNPs to construct a whole Apt-AuNPs probe. In the presence of a target protein, aptamers attempt to specifically bind with them in free solution, which induces a nearly complete dissociation from its complementary sequence and a signal change.

Here, recombinant human erythropoietin-α (rHuEPO-α) is employed as a representative protein to verify the feasibility of our work. Erythropoietin (EPO) belongs to a family of important hematopoietic growth factors, which exert their erythropoiesis production regulation function by promoting the proliferation and differentiation of erythroid progenitor cells, thus maintaining the red blood cell mass at an optimum level [18]. Its highly structurally similar counterpart, recombinant human erythropoietin (rHuEPO) has been widely employed in the clinic for the treatment of anemia associated with renal disease and cancer in the last decade. Currently, immunological assays are the main ways to monitor this drug [19,20], but the batch-to-batch reproducibility and specificity of anti-rHuEPO antibodies is needed to be further improved. The present paper describes a simple signal transduction system for rHuEPO-α. It should be noted that it is not necessary that the specific binding sites of the aptamer for rHuEPO-α be known, and the advantageous features of such a probe mainly come from the competing behavior of the aptamer between its target proteins and cODN which thus should be optimized. Some crucial factors have been examined in detail.

## 2. Experimental Section

### 2.1. Chemicals

The rHuEPO-α (3.89 mg/mL, purity > 98.5%) was provided by SCIPROGEN Bio-pharmaceutical Co. (Shenzhen, China) and diluted to a stock solution of 75 μM containing 0.1% bovine serum albumin (BSA). Hydrogen tetrachloroaurate (III) trihydrate (HAuCl_4_·3H_2_O) was obtained from Alfa Aesar (Ward Hill, MA, USA). The oligonucleotide were synthesized by Sangon Biological Engineering Technology Co. Ltd. (Shanghai, China) and used as received. The 3′-FAM modified aptamer sequence is 5′-TTGAAAGGTCTGTTTTTGGGGTTGGTTTGGGTCAA-FAM-3′, and its complementary oligonucleotide used in this research were listed in Table 1. Sterilized ultrapure water (Milli-Q ultrapure water system, Millipore, Billerica, MA, USA) was used to prepare all of the aqueous solutions. All reagents used in this work were of analytical grade or better.

### 2.2. Synthesis of Citrate Capped AuNPs

Gold nanoparticles were synthesized by reducing tetrachloroauric acid with trisodium citrate [21]. HAuCl_4_·3H_2_O solution (1 mM, 100 mL) was boiled with vigorous stirring in a 250 mL round-bottom flask equipped with a condenser to maintain the reaction mixture at a constant volume. Trisodium citrate (38.8 mM, 10 mL) was added rapidly to the boiling solution, resulting in a color change from pale yellow to dark red, which indicated the formation of AuNPs. The solution was maintained for 15 min at boiling temperature and then removed from the heating mantle. Stirring was continued for another 15 min. For purification, 1.0% SDS (10 μL) was added to the resulting AuNPs solution (1.0 mL), and then centrifuged for 20 min at 14,000 rpm, and after that the AuNPs were resuspended in the ultrapure water. The synthesized AuNPs were characterized by UV-vis spectroscopy (Cary 300, Varian Co., Palo Alto, CA, USA) and transmission electron microscopy (TEM, H7650, Hitachi High-Technologies Co., Tokyo, Japan). The concentration of the AuNPs can be calculated according to Beer’s law using an extinction coefficient of 2.4 × 10^8^ M^−1^ cm^−1^ at 520 nm for the 12 nm AuNPs.

### 2.3. Preparation of cODN Capped AuNPs

The sequences of 5′ thiol-modified cODNs are listed in Table 1. These cODNs were reacted directly with the AuNPs through attachment of the oligo-thiol units onto the AuNPs surface. The AuNPs solutions (1.0 mL) were mixed firstly with 100 μM 5′-thiol-cODNs (30 μL). After reacting for an initial 24 h at 50 °C, the cODN capped AuNPs solution was buffered with a final concentration of 10 mM Tris-HCl and 0.01% SDS, pH 7.4. The mixtures were then equilibrated for 1 h before gradually exposed to 0.3 M NaCl over 48 h. After that the particles were shaken at 100 rpm for an additional 24 h at 50 °C. According to a procedure reported by Mirkin and co-workers [22], this salt “aging” step was of benefit to improve the stability and conjugation efficiency between AuNPs and thiol modified cODN.

To remove all unbound cODNs, the cODN-AuNPs conjugates were centrifuged for 20 min at 14,000 rpm and washed with supernatants. After two wash/centrifuge cycles, the cODN-AuNPs were resuspended separately in the sterile ultrapure water and stored at 4 °C. The sizes of both the citrate capped AuNPs and cODN capped AuNPs were characterized by TEM analysis.

To determine the numbers of cODNs on each AuNPs, a solution of dithiothreitol (DTT, 1.0 M, 100 μL) was used to cleave the Au-S bond and to release all conjugated cODNs from the surface of the AuNPs (12 nM, 100 μL). After overnight incubation at 50 °C, the dissociated cODNs and AuNPs were separated by centrifugation, the absorbance intensity at 260 nm of supernatant were measured to calculate the quantity of displaced ODNs [23] and an average number of ODNs per particle were determined. Our calculation indicated that nearly 80 ODNs were attached to each AuNPs.

### 2.4. Construction of Apt-AuNPs Probe

The obtained cODN capped AuNPs were then hybridized with a 3′-FAM modified aptamer 807-35nt (3′-FAM-Apt). The concentrations of cODN-AuNPs and FAM-Apt were maintained at 1:20 ratio constantly. In a typical experiment, the FAM-Apt (0.12 μM) was allowed to hybridize with cODN capped AuNPs (400 μL, 6 nM) of different complementary recognition sequences in the selection buffer (20 mM Tris-HCl, 140 mM NaCl, 5 mM MgCl_2_ and 5 mM KCl at pH 7.4) for 10 h at 4 °C after heating at 90 °C for 5 min, and then equilibrated with 0.1% BSA for 4 h at 4 °C before using. The BSA-blocked step can prevent nonspecific binding with any possible interfering proteins. After hybridization and blocking, the Apt-AuNPs probe, *i.e.*, the bioconjugate of cODN-AuNPs and FAM-Apt was formed.

### 2.5. Fluorescence Assays

Different amounts of rHuEPO-α (10.0 μL) were added to the Apt-AuNPs solution which had been dispersed in the selection buffer and maintained at room temperature for 5 h to reach a binding equilibrium. Before collecting fluorescence spectra (LS55 spectrofluorometer, Perkin Elmer, Waltham, MA, USA), each of solutions were then transferred into quartz cuvettes. Both excitation and emission slit widths were set at 7 nm and an excitation wavelength of 495 nm was used throughout the experiments.

### 2.6. Competitive Assay

Different interfering proteins including HSA, IgG, transferrin, globin, lysozyme and cytochrome C were used to examine the specificity of the assay. Each of the interfering protein (1 μM) were added to the rHuEPO-α sensing system which contained Apt-AuNPs (400 μL, 6 nM) and rHuEPO-α (100 nM) in the selection buffer, and allowed to standing at room temperature for about 5 h, then the solution was transferred into a quartz cuvette to collect the fluorescence spectrum.

## 3. Results and Discussion

### 3.1. Ligand Replacement on the Surface of AuNPs

**Figure 1.**
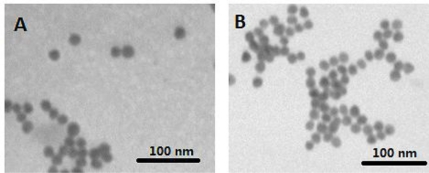
TEM images of citrate capped AuNPs (**A**) and cODN capped AuNPs (**B**).

The characteristics of citrate and cODN capped AuNPs have been compared based on their shapes and surface plasma peaks. Both citrate and cODN capped AuNPs are well-dispersed and have the same average diameter of 12 nm (Figure 1), indicating that the replacement of citrate by cODN does not affect the diameter of AuNPs and its state of dispersion. The surface plasma peak of the latter shows a slight red-shift from 520 nm to 522 nm. This can be attributed to the replacement of citrate by cODN on the surface of AuNPs. The cODN capped AuNPs aqueous solution is quite stable, and no aggregation is observed, even after 6 months storage at 4 °C.

### 3.2. Systematic Optimization on Signal-On Mode of Apt-AuNPs Probe

Recently, our group has successfully evolved single stranded (ss) DNA aptamers with high specificity and affinity for rHuEPO-α [23], which would provide an alternative and ideal recognition module for rHuEPO-α in analytical science. Some detection methods based on this anti-rHuEPO-α aptamer have been developed and proved its feasibility [24–26].

**Scheme 1.**
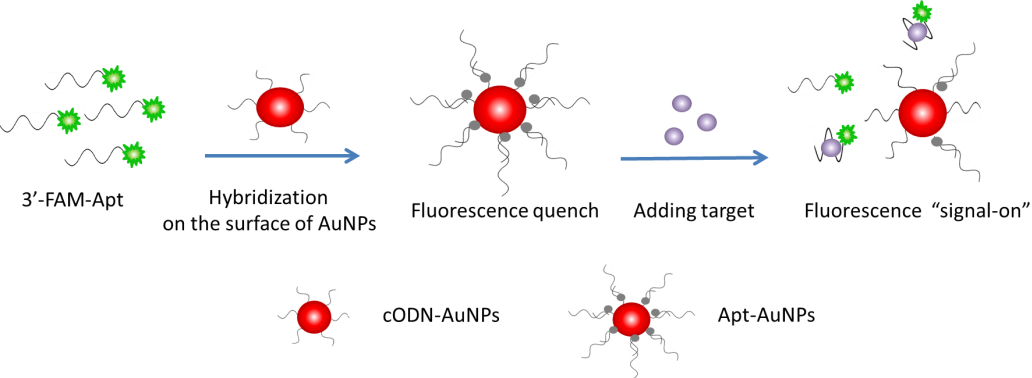
Schematic of Apt-AuNPs probe for rHuEPO-α detection, which is based on a modulation of the FRET between FAM-Apt and cODN-AuNPs.

In this work, an aptamer based AuNPs (Apt-AuNPs) probe is designed to transduce a molecular binding event into a fluorescence signal. The Apt-AuNPs probe consists of a gold core and a dense monolayer of chemisorbed cODN sequences, which are hybridized with the 3′-FAM labeled aptamer oligonucleotides (FAM-Apt). The fluorescence of FAM is efficiently quenched by the AuNPs through FRET [27,28] after hybridization, as depicted in Scheme 1. In the presence of rHuEPO-α target, the assembled aptamer is induced to form a folded structure to fit the binding event, and the base-pairing behavior between FAM-Apt and the cODN-AuNPs is disrupted, which causes FAM-Apt to be released from the surface of cODN-AuNPs and the fluorescence to be restored.

The key considerations to achieve the best sensitivity of this assay are both elimination of the background fluorescence and regeneration of the inherent fluorescence of Apt-AuNPs in the presence of rHuEPO-α. We describe a series of experiments to systematically evaluate the variables that relate to the sensitivity for the target protein. These variables include spacer length, the complementary length of cODN linked to AuNPs as well as hybridization site between FAM-Apt and cODN-AuNPs. The relative molar ratio of cODN-AuNPs and FAM-Apt was held constant in this work in order to minimize any differences in fluorescence intensity occurring from changes in the DNA coverage.

### 3.3. Length of Poly(dA) Spacer

When fluorescently tagged ODNs are located near metal surfaces, their emission intensity is impacted by both electromagnetic effects (*i.e.*, quenching and/or enhancement of emission) and the structures of ODNs. Therefore, the presence of the poly(dA) spacer is important in stabilizing gold cores and increasing Apt-AuNPs hybridization efficiency by maintaining the target recognition sequences far enough from the particle surface.

**Figure 2.**
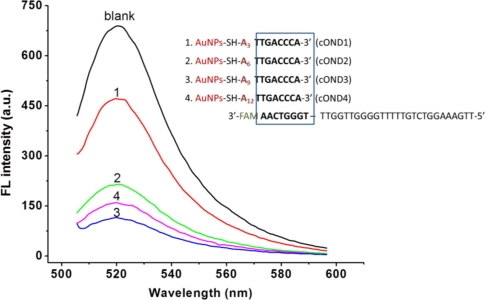
Effects of spacer length on FRET efficiency after FAM-Apt hybridizing with cODN-AuNPs. The concentration of FAM-Apt is 120 nM, and blank represents only FAM-Apt existed. Curves 1, 2, 3 and 4 represent the fluorescence of four kinds of Apt-AuNPs containing 3, 6, 9 or 12 nt spacers, respectively.

To investigate the effect of the spacer length on the fluorescence of Apt-AuNPs probe, four cODN-AuNPs (functionalized with cODN1 to 4, Table 1) containing 3, 6, 9 or 12 nt spacers have been designed to hybridize with FAM-Apt in 8 bp length from its 3′ end, respectively. As shown in Figure 2, the fluorescence intensity in the cODN3-AuNPs and FAM-Apt bioconjugate is the lowest one with a metal-fluorophore distance of 9 nt (3.2 nm if fully extended) from the surface. Short spacer length (3 and 6 nt) might cause low hybridization efficiency due to the steric hindrance of AuNPs, on the other hand, distance greater than 12 nt would lead to an insufficient FRET efficiency, and both situations would result in the decay of the signal-to-noise ratio because of the relative high fluorescence background. Herein, we choose spacer dA of 9 nt as the most suitable distance between cODN-AuNPs and the FAM-Apt for all the subsequent experiments.

### 3.4. Hybridization Position

**Figure 3.**
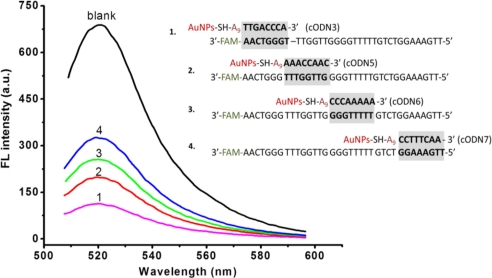
Effect of hybridization sites between cODN-AuNPs and FAM-Apt on the background fluorescence intensity of Apt-AuNPs. The concentration of FAM-Apt is 120 nM, and blank represents only FAM-Apt existed. Curves 1, 2, 3 and 4 represent the fluorescence of four kinds of Apt-AuNPs having different hybridization sites of 8 bp.

In such an Apt-AuNPs probe, FAM-Apt was partially hybridized with cODN-AuNPs for quenching the fluorescence. We carried out experiments to explore the effect on fluorescence intensity of the position of hybridization of FAM-Apt with the cODN-AuNPs. Four cODNs on AuNPs having different hybridization sites with FAM-Apt from its 3′ end to 5′end were investigated. As illustrated in Figure 3, the bioconjugate of cODN3-AuNPs and FAM-Apt whose hybridization occurs in the site near the 5′ end of the aptamer, displays maximum FRET efficiency compared to the other three Apt-AuNPs functionalized with cODN5, cODN6 and cODN7, respectively (Table 1). This result indicates that the efficiency of energy transfer is strongly dependent on the distance between FAM (donor) and AuNPs (acceptor). To eliminate the background fluorescence, we chose cODN3 as the best complement sequence.

### 3.5. Hybridization Length between cODN-AuNPs and FAM-Apt

To find a suitable length of complementary ODN which can offer low background fluorescence after hybridizing with FAM-Apt, while FAM-Apt can provide an easy structural switching when the target is added, we have examined a series of cODN-AuNPs with different hybridization lengths ranging from 5 to 8 bp (cODN8, 9, 10 and 3 in Table 1).

**Figure 4.**
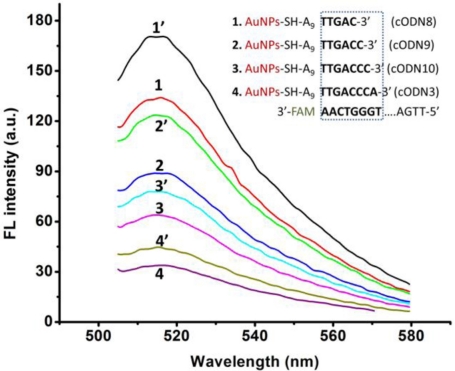
Effect of different hybridization length on the FRET efficiency of Apt-AuNPs and the fluorescence recovery of it in the presence of 60 nM rHuEPO-α. Curves 1 (1′), 2 (2′), 3 (3′) and 4 (4′) represent the fluorescence intensity of the formed Apt-AuNPs before and after binding with rHuEPO-α (60 nM), respectively.

As shown in Figure 4, the fluorescence intensity of Apt-AuNPs increases slightly when the hybridization length changes from 8 to 5 bp, and the specific competition ability of FAM-Apt is improved to some extent. The results show that Apt-AuNPs probe with 5 or 6 bp hybridization (named cODN8-AuNPs, cODN9-AuNPs, respectively) provides a comparably highly sensitive signal for the determination of rHuEPO-α, but cODN9-AuNPs and FAM-Apt conjugate have lower fluorescence background than that of cODN8-AuNPs. A possible explanation is a DNA hybrid of more than half a helix (5 bp) would generate a more tight duplex structure in Apt-AuNPs, which impede rHuEPO-α from competing with such a hybrid for FAM-Apt.

**Figure 5.**
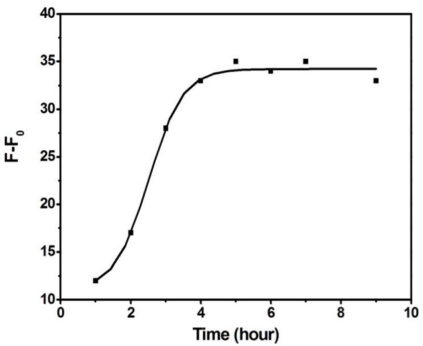
The fluorescence recovery (F-F_0_) of Apt-AuNPs probe in the presence of 60 nM rHuEPO-α.

Although the fluorescence background is lower in the cases of cODN-AuNPs consisting of 7 or 8 bp (cODN 10 and 3), the specific competition ability of target protein is obviously diminished. Here we choose cODN9 of a hybridization length of 6 bp as the most suitable length, and employ it to perform the quantitative assay. We also examined here the binding time equilibrium between rHuEPO-α and the aptamer. As illustrated in Figure 5, the competitive binding reached equilibrium after 5 h, and the luminescence signal remained stable for more than 10 h.

### 3.6. Determination of Target rHuEPO-α Using Apt-AuNPs Probe

**Figure 6.**
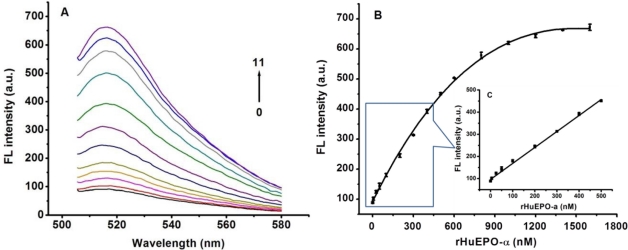
(**A**) Fluorescence “signal-on” measurement of rHuEPO-α. The concentration of rHuEPO-α is 0, 5, 25, 50, 100, 200, 300, 400, 600, 800, 1,000 and 1,600 nM from line 0 to 11, respectively; (**B**) Plot of fluorescence intensity (*F*) of the Apt-AuNPs probe *vs.* the concentration of rHuEPO-α; (**C**) The linear relationship between *F* and the rHuEPO-α concentration. Each point reflects the average of three independent experiments. Error bars indicate standard deviations.

Under the optimized conditions, the performance of the Apt-AuNPs based “signal-on” method for detecting rHuEPO-α was further evaluated. As shown in Figure 6, an increase in rHuEPO-α concentration leads to an obvious recovery of the fluorescence emission of the Apt-AuNPs consisting of cODN9-AuNPs and FAM-Apt. A linear relationship between the change in the relative fluorescence intensity (*F*, where *F* and *F*_0_ are the peak fluorescence intensity obtained with the analyte and the blank sample, respectively) of this sensing system and the concentration of rHuEPO-α from 0 to 500 nM was observed (*F* = 96.4 + 0.721C_rHuEPO-__α_, *R* = 0.997). A LOD of 0.92 nM was calculated according to the equation of LOD = 3SD_blank_/k (where k represents the slope of linear curve, the repeated number of blank is 10).

**Figure 7.**
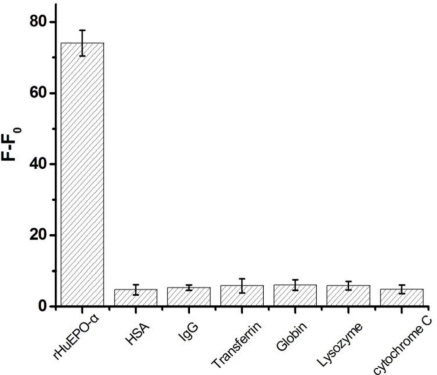
Specificity of the Apt-AuNPs probe, which is reflected by the fluorescence responses (F-F_0_) of the Apt-AuNPs probe in the presence of 100 nM rHuEPO-α in the presence of 1.0 μM of different interfering proteins. Each point reflects the average of three independent experiments. Error bars indicate standard deviations.

To address the issue of selectivity of this sensing system, we have also examined various common co-existing interfering proteins. It is found that addition of such a protein does not generate a significant increase in fluorescent signal (Figure 7). These results are consistent with a selective target binding ability of the aptamer.

## 4. Conclusions

In this work, detection of rHuEPO-α relies upon the competitive binding event between the target molecule and the aptamer-cODN hybrid chemisorbed on AuNPs. This Apt-AuNPs probe provides a way to fluorescently monitor the amount of rHuEPO-α at nM level in a simple manner and shows high selectivity compared to other common proteins. Despite the benefits of this method, could was still unsuitable for clinic practice (the concentration of rHuEPO-α is pM in urine and blood). Further research should be focused on devising or adopting some pre-enrichment method and combined it with this Apt-AuNPs probe, which would then be expected to be a potential and powerful tool in biomonitoring rHuEPO-α in clinical diagnostics.

## Figures and Tables

**Table 1.** The ssDNA sequence used in the experiments.

**ssDNA**	**Sequence(5′→3′)**
**cODN1**	5′-SH-AAA TTG ACC CA-3′
**cODN2**	5′-SH-AAA AAA TTG ACC CA-3′
**cODN3**	5′-SH-AAA AAA AAA TTG ACC CA-3′
**cODN4**	5′-SH-AAA AAA AAA AAA TTG ACC CA-3′
**cODN5**	5′-SH-AAA AAA AAA AAA CCA AC-3′
**cODN6**	5′-SH-AAA AAA AAA CCC AAA AA-3′
**cODN7**	5′-SH-AAA AAA AAA CCT TTC AA-3′
**cODN8**	5′-SH-AAA AAA AAA TTG AC-3′
**cODN9**	5′-SH-AAA AAA AAA TTG ACC-3′
**cODN10**	5′-SH-AAA AAA AAA TTG ACC C-3′

## References

[1] Xiao Y., Patolsky F., Katz E., Hainfeld J.F., Willner I. (2003). “Plugging into enzymes”: Nanowiring of redox enzymes by a gold nanoparticle. Science.

[2] Tkachenko A.G., Xie H., Coleman D., Glomm W., Ryan J., Anderson M.F., Franzen S., Feldheim D.L. (2003). Multifunctional gold nanoparticle-peptide complexes for nuclear targeting. J. Am. Chem. Soc.

[3] Chithrani B.D., Ghazani A.A., Chan W.C.W. (2006). Determining the size and shape dependence of gold nanoparticle uptake into mammalian cells. Nano Lett.

[4] Stoeva S.I., Lee J.S., Smith J.E., Rosen S.T., Mirkin C.A. (2006). Multiplexed detection of protein cancer markers with biobarcoded nanoparticle probes. J. Am. Chem. Soc.

[5] Lee J.S., Lytton-Jean A.K.R., Hurst S.J., Mirkin C.A. (2007). Silver nanoparticle-oligonucleotide conjugates based on DNA with triple cyclic disulfide moieties. Nano Lett.

[6] Dubertret B., Calame M., Libchaber A.J. (2001). Single-mismatch detection using gold-quenched fluorescent oligonucleotides. Nat. Biotechnol.

[7] Cao Y.W.C., Jin R.C., Mirkin C.A. (2002). Nanoparticles as labels for biodiagnostic rsearch. Science.

[8] Nam J.M., Thaxton C.S., Mirkin C.A. (2003). Nanoparticle-based bio-bar codes for the ultrasensitive detection of proteins. Science.

[9] Giljohann D.A., Seferos D.S., Daniel W.L., Massich M.D., Patel P.C., Mirkin C.A. (2010). *In vitro* selection of structure-switching signaling aptamers. Angew. Chem. Int. Ed.

[10] Ellington A.D., Szostak J.W. (1990). *In vitro* selection of RNA molecules that bind specific ligands. Nature.

[11] Tuerk C., Gold L. (1990). Systematic evolution of ligands by exponential enrichment: RNA ligands to bacteriophage T4 DNA polymerase. Science.

[12] Breaker R.R. (1997). DNA enzymes. Nat. Biotechnol.

[13] Chen J.W., Liu X.P., Feng K.J., Liang Y., Jiang J.H., Shen G.L., Yu R.Q. (2008). Detection of adenosine using surface-enhanced Raman scattering based on structure-switching signaling aptamer. Biosens. Bioelectron.

[14] Nutiu R., Li Y.F. (2005). *In vitro* selection of structure-switching signaling aptamers. Angew. Chem. Int. Ed.

[15] Huang C.C., Chiu S.H., Huang Y.F., Chang H.T. (2007). Aptamer-functionalized gold nanoparticles for turn-on light switch detection of platelet-derived growth factor. Anal. Chem.

[16] Zheng D., Seferos D.S., Giljohann D.A., Patel P.C., Mirkin C.A. (2009). Aptamer nano-flares for molecular detection in living cells. Nano Lett.

[17] Zhang J., Wang L., Zhang H., Boey F., Song S., Fan C. (2010). Aptamer-based multicolor fluorescent gold nanoprobes for multiplex detection in homogeneous solution. Small.

[18] Choi D., Kim M., Park J. (1996). Erythropoietin: Physico- and biochemical analysis. J. Chromatogr. B.

[19] Abellan R., Ventura R., Pichini S., Remacha A.F., Pascual J.A., Pacifici R., Giovannandrea R.D., Zuccaro P., Segura J. (2004). Erythropoietin (EPO) as an indirect biomarker of recombinant human EPO misuse in sport. J. Pharm. Biomed. Anal.

[20] Yu B., Cong H.L., Liu H.W., Li Y.Z., Liu F. (2005). Ionene-dynamically coated capillary for analysis of urinary and recombinant human erythropoietin by capillary electrophoresis and online electrospray ionization mass spectrometry. J. Sep. Sci.

[21] Storhoff J.J., Elghanian R., Mucic R.C., Mirkin C.A., Letsinger R.L. (1998). One-pot colorimetric differentiation of polynucleotides with single base imperfections using gold nanoparticle probes. J. Am. Chem. Soc.

[22] Hill H.D., Millstone J.E., Banholzer M.J., Mirkin C.A. (2009). The role radius of curvature plays in thiolated oligonucleotide loading on gold nanoparticles. ACS Nano.

[23] Zhang Z., Guo L., Guo A.T., Xu H., Tang J.J., Guo X.J., Xie J.W. (2010). *In vitro* lectin-mediated selection and characterization of rHuEPO-α-binding ssDNA aptamers. Bioorg. Med. Chem.

[24] Zhang Z., Guo L., Tang J., Guo X., Xie J. (2009). An aptameric molecular beacon-based “Signal-on” approach for rapid determination of rHuEPO-alpha. Talanta.

[25] Tang J., Guo L., Shen R., Yu T., Xu H., Liu H., Ma X., Xie J.W. (2010). Quantification of rHuEPO-α by magnetic beads-based aptameric real-time PCR assay. Analyst.

[26] Shen R., Guo L., Zhang Z.Z., Meng Q.W., Xie J.W. (2010). Determination of rHuEPO-α by an aptamer-based affinity capillary electrophoresis-laser induced fluorescence detection method. J. Chromatogr. A.

[27] Otsuka H., Akiyama Y., Nagasaki Y., Kataoka K. (2001). Quantitative and reversible lectin-induced association of gold nanoparticles modified with α-Lactosyl-ω-mercapto-poly(ethylene glycol). J. Am. Chem. Soc.

[28] Dulkeith E., Morteani A.C., Niedereichholz T., Klar T.A., Feldmann J., Levi S.A., van Veggel F.C., Reinhoudt D.N., Moller M., Gittins D.I. (2002). Fluorescence quenching of dye molecules near gold nanoparticles: Radiative and nonradiative effects. Phys. Rev. Lett.

